# Syndrome de Leriche chez une jeune femme de 20 ans: à propos d’un cas

**DOI:** 10.11604/pamj.2021.39.181.30484

**Published:** 2021-07-07

**Authors:** Leila Noureddine, Daoud Bentaleb, Keltoum Boumlik, Mouna Sabiri, Ghizlane Lembarki, Samira Lezar, Samia Elmanjra, Fatiha Essodegui

**Affiliations:** 1 Service Central de Radiologie, Centre Hospitalier Universitaire Ibn Rochd de Casablanca, Casablanca, Maroc

**Keywords:** Syndrome de Leriche, angioscanner, occlusion, aorto-iliaque, à propos d’un cas, Leriche syndrome, angioscan, occlusion, aortoiliac, case report

## Abstract

Le syndrome de Leriche, ou syndrome d´oblitération aorto-iliaque, est une entité particulière d´artériopathie oblitérante des membres inférieurs qui consiste en une occlusion thrombotique du carrefour aorto-iliaque. Nous rapportons le cas d´une patiente âgée de 20 ans, sans antécédents pathologiques particuliers connus, qui s´est présentée avec un tableau d´ischémie aiguë des deux membres inférieurs. L´écho Doppler des membres inférieurs a retrouvé une diminution globale des flux artériels sans visualisation de matériel endoluminal. Ceci a motivé la réalisation d´un angioscanner des membres inférieurs qui a objectivé une thrombose artérielle étendue de l´aorte abdominale aux artères iliaques externes bilatérales. La patiente a par la suite bénéficié d´un pontage aorto-bi-iliaque avec une bonne évolution, ainsi que d´une démarche diagnostique étiologique à la recherche d´un éventuel terrain thrombogène.

## Abstract (en)

Leriche syndrome or aortoiliac occlusive disease is a particular obliterative arterial disease of the lower limbs, consisting of thrombotic occlusion of the aortoiliac junction. We here report the case of a 20-year-old female patient with no particular past medical history, presenting with acute lower limb ischemia. Lower extremity echo Doppler showed overall decrease in arterial blood flow without visualization of endoluminal material. Lower limb angioscaner showed arterial thrombosis of the abdominal aorta extended to bilateral external iliac arteries. The patient subsequently underwent aortic-bi-iliac bypass surgery with good evolution as well as assessments for possible thrombogenic mechanism.

## Introduction

Le syndrome de Leriche, ou syndrome d´oblitération du carrefour aorto-iliaque, est une entité particulière liée à l´occlusion thrombotique de la terminaison de l´aorte abdominale. C´est une affection assez rare, à la clinique parfois trompeuse, et dont l´aspect à l´imagerie est typique, notamment sur un angioscanner [1,2].

## Patient et observation

**Informations du patient et résultats cliniques**: il s´agit d´une patiente âgée de 20 ans, sans antécédents pathologiques particuliers connus, ayant présenté 10 jours avant son admission un tableau d´ischémie mésentérique révélé par des vomissements et des douleurs abdominales aiguës. A cette symptomatologie s´est ajouté, 2 jours avant son admission, un tableau d´ischémie aiguë des deux membres inférieurs avec un début de nécrose des orteils.

**Figure 1:**
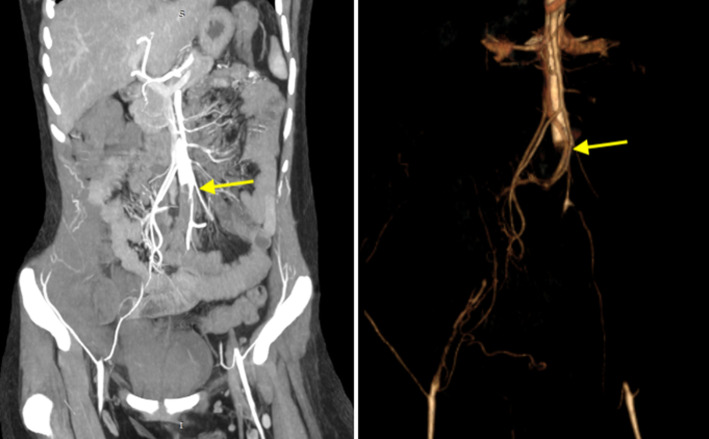
angioscanner des membres inférieurs avec une étude des vaisseaux pelviens et abdominaux, qui a montré une occlusion artérielle totale étendue de l’aorte abdominale aux artères iliaques externes bilatérales (flèches)

**Évaluation diagnostique**: devant ce tableau, un écho Doppler des deux membres inférieurs a été réalisé chez elle, objectivant une diminution diffuse des flux artériels, contrastant avec l´absence de visualisation de matériel endoluminal. Ces données ont motivé la réalisation d´un angioscanner des membres inférieurs avec une étude des vaisseaux pelviens et abdominaux, qui a montré une occlusion artérielle totale étendue de l´aorte abdominale aux artères iliaques externes bilatérales (Figure 1).

**Intervention thérapeutique et suivi**: la patiente a par la suite bénéficié d´un traitement chirurgical (pontage aorto-bi-iliaque) avec une bonne évolution, ainsi que d´une hospitalisation en médecine interne pour diagnostic étiologique, à la recherche d´un éventuel terrain prédisposant tel qu´un syndrome des anti-phospholipides.

## Discussion

Décrit pour la première fois par Robert Graham en 1814 à l´Infirmerie Royale de Glasgow puis par le chirurgien français René Leriche en 1923 [3], le syndrome d´occlusion de l´aorte abdominale à sa bifurcation ainsi que des artères iliaques primitives, qui a depuis pris le nom de « syndrome de Leriche », est une forme rare et particulière d´artériopathie oblitérante des membres inférieurs [1]. Sur le plan clinique, le syndrome de Leriche comprend: une claudication de cuisse ou de hanche, une hypotrophie musculaire, une hypopulsatilité fémorale, et chez l´homme, une impuissance sexuelle dans un quart des cas [4]. Il peut être également révélé par des tableaux trompeurs, simulant par exemple une sciatique [5], ou par une symptomatologie d´apparition aiguë, comme dans le cas de notre patiente. Sur le plan artériel, il s´agit d´une occlusion chronique complète de l´aorte terminale. Elle touche plus souvent les sujets de sexe masculin avec un âge moyen au moment du diagnostic aux alentours de 50 ans [6]. La constatation de ce syndrome chez un sujet jeune doit faire rechercher un terrain particulier thrombogène, tel qu´un syndrome des anti-phospholipides.

Les lésions caractéristiques du syndrome de Leriche sont de quatre types: a) sténoses isolées des iliaques primitives, b) lésions plus ou moins étendues de la bifurcation aortique n´intéressant que la terminaison de l´aorte et l´origine des iliaques primitives, c) lésions étendues de l´aorte abdominale et des iliaques, comme dans notre cas, d) occlusion complète de l´aorte sous-rénale [6]. L´imagerie diagnostique du syndrome de Leriche et des occlusions artérielles des membres inférieurs en général repose d´abord sur l´angiographie artérielle, qui reste le gold-standard, de par son étude en haute résolution de tout l´arbre vasculaire du membre inférieur et la possibilité de réaliser des gestes interventionnels. Elle reste cependant invasive; et l´angioscanner de l´aorte abdominale et des membres inférieurs en constitue ainsi une alternative avantageuse de par sa nature non invasive, ainsi que la possibilité d´étudier également toute la région avoisinante à la recherche d´autres pathologies chez des sujets aux nombreuses comorbidités [7].

L´angioscanner nous permet de poser le diagnostic de la thrombose de l´aorte abdominale, de déterminer sa nature occlusive totale ou partielle et d´évaluer son étendue notamment aux iliaques primitives, ainsi que la présence et l´importance d´une éventuelle circulation collatérale associée -dont il importe de déterminer l´origine. Tous ces éléments permettent au clinicien de planifier une prise en charge adéquate pour le patient [3]. Ses principales limites sont sa résolution verticale limitée, qui peut sous-estimer ou même passer à côté d´une sténose courte, ainsi que la présence de plaques calcifiées qui allongent le temps de post-traitement et peuvent gêner l´analyse correcte des parois vasculaires [2,8]. Le traitement du syndrome de Leriche est essentiellement chirurgical à type de pontage aorto-bi-iliaque ou aorto-fémoral, avec une faible morbi-mortalité et une excellente perméabilité vasculaire à distance. L´angioplastie transluminale en kissing (avec ou sans thrombolyse préalable) prend également une place de plus en plus importante en première intention, avec de bons résultats immédiats [6].

## Conclusion

L´angioscanner constitue l´examen de choix dans le diagnostic du syndrome de Leriche dans la mesure où il permet une étude vasculaire de haute résolution de l´aorte et des artères des membres inférieurs et de déterminer l´étendue de l´occlusion ainsi que l´origine et l´importance de la circulation collatérale. Ceci, dans l´optique de planifier le traitement -qui est le plus souvent chirurgical- afin d´améliorer le pronostic fonctionnel du patient.
